# Health workers’ experiences with the Safe Delivery App in West Wollega Zone, Ethiopia: a qualitative study

**DOI:** 10.1186/s12978-019-0725-6

**Published:** 2019-05-09

**Authors:** Camilla Faldt Thomsen, Anne Marie Frøkjær Barrie, Ida Marie Boas, Stine Lund, Bjarke Lund Sørensen, Feyisa Gudeta Oljira, Britt Pinkowski Tersbøl

**Affiliations:** 1 Department of Public Health, Global Health Section, University of Copenhagen, Øster Farimagsgade 5, 1014 Copenhagen K, Denmark; 2 Maternity Foundation, Sortedam Dossering 81, 2100 Copenhagen Ø, Denmark; 3 Department of Pediatrics, Global Health Unit, Rigshospitalet, Blegdamsvej 9, 2100 Copenhagen Ø, Denmark; 4 Centre for Innovative Medical Technology, University of Southern Denmark, Campusvej 55, 5230 Odense M, Denmark; 5 Maternity Foundation, P. O. Box 158, Gimbie, Oromia Ethiopia

**Keywords:** Health workers, Maternal health, Perinatal health, Childbirth, mHealth, Mobile phone, Smartphone, Ethiopia, E-health, Digital health

## Abstract

**Background:**

Health workers in many low-income countries are not adequately trained to deliver pregnant women safely. In response to this, the Safe Delivery App (SDA) has been developed, which provides animated clinical instruction videos in basic emergency obstetric and neonatal care. The SDA aims to improve knowledge and skills of health workers located in the periphery of the health system in order to improve quality of care and potentially save the lives of mothers and newborns. The objective of this qualitative study was to explore the users’ experiences with using the SDA and in which ways the SDA influences their work situation and their perceived ability to conduct safe deliveries.

**Methods:**

Eleven focus group discussions and four individual interviews were conducted with a total of 56 midwives, nurses and health extension workers from five districts in West Wollega Zone, Oromiya region of Ethiopia. The data further include observations and informal conversations. All interviews were recorded, transcribed verbatim, checked for corrections and analysed using systematic text condensation.

**Results:**

The findings indicate that health workers perceive the SDA as a useful tool, which helps them memorize and update knowledge and skills, and improves their confidence. User patterns follow the relevancy of the tool to the health workers’ work situation - those who conduct many deliveries have more often used the app in emergency situations, whereas those who conduct few deliveries more often use it to improve their knowledge and to provide health education to pregnant women. Thus, the SDA is used in varying ways depending on internal and external factors such as own competencies, availability of equipment and frequency of births attended. Health workers experienced that community members showed more recognition and trust in their abilities and ascribed this to their increased confidence in assisting in deliveries. The increased recognition from communities may also be associated to a medical technology.

**Conclusion:**

The health workers perceive the SDA as having improved their ability to manage complications during childbirth and have gained increased recognition and trust from the communities.

## Plain English summary

Maternal and perinatal mortality remain a significant challenge in many low- and middle-income countries. To empower health workers to provide a safer birth for mothers and newborns, a smartphone application referred to as ‘The Safe Delivery App’ (SDA) has been developed. The SDA aims to improve knowledge and skills of health workers located in the periphery of the health system by providing visual animated instruction videos and guiding steps in basic emergency obstetric and neonatal care. This study sought to explore Ethiopian health workers’ experiences with using the SDA and how the SDA influences their work situation and their perceived ability to conduct safe deliveries. Data was collected through qualitative interviews and focus group discussions conducted with a total of 56 Ethiopian health workers. The findings indicate that the health workers perceive the SDA as a useful tool, which helps them memorize and update knowledge and skills, improves their confidence and makes them feel better able to deliver quality care. Consequently, the health workers experience more trust and respect from the communities. The app is used in multiple ways depending on the relevance of the tool to the health workers’ work situation. Those who conduct many deliveries more often state that they use the app in clinical situations, whereas those who conduct few deliveries more often use it to improve their knowledge and to provide health education to pregnant women.

## Background

In spite of increased global efforts to place maternal and child health on the political agenda, maternal and perinatal mortality still remain a significant challenge in many low- and middle-income countries. Every year an estimated 303,000 maternal deaths [1], 2,7 million neonatal deaths [2] and 2,6 million stillbirths occur [3] though the majority of these deaths could have been prevented, had the women had access to the recommended quality care during pregnancy, childbirth and post-partum period. Maternal deaths have devastating consequences for the families’ wellbeing. In addition, millions of women, who survive childbirth, suffer from severe and lifelong injuries. Despite the overall decrease in mortality among children younger than 5 years, the proportion of deaths that occur in the neonatal period is increasing [4] and there is an urgent need to focus on the large burden of stillbirths, which has not been prioritised in the Millennium Development Goals [5]. Hence, there is an urgent need for global action to improve maternal and neonatal health in low- and middle-income settings.

Low utilisation of delivery services and an inadequate level of quality delivery care are significant contributors to the high rate of maternal and perinatal mortality and morbidity. In Ethiopia, approximately 412 maternal deaths per 100,000 live births and 48 infant deaths per 1000 live births occurred between 2011 and 2016 [6]. Ethiopia is, thus, one of the countries in the world with the highest number of stillbirths, perinatal and maternal deaths [5]. Despite this, only 26% of births are delivered at a health facility [6]. The majority of perinatal deaths are caused by preterm birth complications, intrapartum asphyxia and infections [5, 7] and the leading direct causes of maternal mortality are haemorrhage, hypertensive disorders, and sepsis [8]. Therefore, there is an urgent need to ensure access to basic emergency obstetric and neonatal care not only by providing access to health facilities but also by improving health workers’ ability to manage obstetric and neonatal emergencies [9].

Within the last decades, there has been a substantial increase in the ownership of mobile phones in low-income countries [10]. Using mobile phones for health (mHealth) hold a significant potential to improve health care, as they provide an opportunity to increase access to and share information despite long distances [11]. However, the evidence for integrating mHealth technologies to improve maternal health care in low-income settings is limited [12]. Previous research has primarily focused on using mHealth technologies to improve health awareness among pregnant women in order to increase service utilization [13–16], or as a way for health workers to collect data [17–21] or communicate with other actors in the health care system [22–24]. Only few studies have focused on using mobile phones to improve knowledge and skills of health workers working with maternal and neonatal care in low- and middle income settings [25, 26]. Among these studies, a controlled intervention study by Nilsson et al. (2014) compared two training methods for postpartum haemorrhage (PPH) (interactive hands-on training vs. a non-interactive training video) among 27 senior nursing students at a secondary health care centre in Kenya. The training methods were evaluated through structured observations of a standardized scenario before and after training and showed increase in performance scores in both groups with no significant difference between the two groups [26].

Maternal and newborn mortality and morbidity rates call for innovative solutions to support and strengthen conventional interventions. The Danish NGO, Maternity Foundation, the University of Copenhagen and the University of Southern Denmark have developed an instructive and educative mHealth technology referred to as the Safe Delivery App (SDA). The purpose of the app is to improve knowledge and skills on management of complicated deliveries among health workers located in peripheral health facilities in low-income settings and thereby improve the quality of life-saving care. For a tool such as the SDA to fulfil its aim, it is essential that the intended users experience the tool as relevant and meaningful, and as adding value to their everyday work. A previous study of the same intervention examined the effect of the SDA on the knowledge and skills of health care workers in neonatal resuscitation and perinatal mortality [25]. Knowledge scores were assessed using a key feature questionnaire and technical skills were assessed using simulated scenarios evaluated by independent and trained midwives. The study showed that the knowledge and skill level in neonatal resuscitation increased significantly 6 and 12 months from baseline among health workers in the intervention group [25]. Having documented this, it is important to document the user’s experience of this tool. This study therefore examines how health workers experience the SDA in the context of their specific working conditions and explores in what ways the SDA influences Ethiopian midwives, nurses and health extension workers’ (HEWs) perception of their work situation and ability to conduct safer deliveries.

## Methods

### Study setting

The study was conducted in the five districts Gimbie, Haru, Nole Kaba, Genji and Homa located in West Wollega Zone in the Oromiya region of Ethiopia. All five districts are part of the Ethiopian highlands characterized by steep hills, poorly constructed roads and long distances between kebeles (villages) and health facilities. The total population of the five districts comprise more than 400,000 people of which the majority live in rural areas and are engaged in agricultural work. There are two hospitals in Gimbie town (one private faith-based and one governmental) and a total of 12 health centres and 116 health posts in the five districts.

### Study design

This qualitative study was conducted in conjunction with a large cluster-randomized clinical trial investigating the influence of the SDA on perinatal mortality, postpartum haemorrhage and the knowledge and skills level of the 176 midwives, nurses and HEWs included in the overall study [25]. With 73 health facilities as the unit of randomization, the study participants were divided into an intervention group and a control group. The intervention group received a smartphone with the SDA installed and went through a 1-day training course to learn how to use the phone and the app. Both groups were provided with a basic delivery kit including among other things soap, sterile gloves, gauze, a self-inflating mask and bag for neonatal resuscitation, and misoprostol or oxytocin for prevention and treatment of PPH. As HEWs are not certified to use e.g. suturing material and IV equipment, the Ethiopian Health Authorities did not allow the distribution of the full delivery kit to HEWs, thus e.g. suturing material and IV equipment was only distributed to midwives and nurses. A total of 56 participants were included in the qualitative study of which seven HEWs participated at both baseline and follow-up. Five FGDs were conducted at baseline and six FGDs at follow-up. As a supplement, we interviewed two health workers who had used the app frequently, and two health workers who hardly used it at all to get an indication of what might lead to very different use patterns. Individual interviews were conducted as a supplement to gain in-depth information about how the app was perceived. The follow-up data collection further included observations and informal conversations.

### The safe delivery app

The version of the SDA that was tested in this study^1^ contains four animated videos with instructions on active management of third stage of labour, management of PPH, manual removal of the placenta, and neonatal resuscitation. The clinical content was developed following international WHO guidelines but focusing on key essential lifesaving interventions. Selection of key features was selected in a standardized Delphi process where international clinical experts scored key features according to relevance. Film manuscripts and draft animations was hereafter developed and approved by the Delphi groups. The app offers the opportunity to watch the entire animation video (5–7 min long) as well as choosing to see a specific procedural step of each film. The app also contains a catalogue with essential drugs and equipment. To stimulate use, the app sends weekly notifications with quiz questions and a direct link to the films where the information to answer the question is found. The SDA can be downloaded in different language versions free of charge.

### Participants

The study participants included 36 HEWs working at health posts and 19 midwives/nurses working at health centres or hospitals. The included HEWs are all women, who have completed at least a grade 10 educational level and consequently attended a one-year certificate training course covering 16 health topics of which one module includes training in safe and clean delivery [27]. HEWs are assigned in pairs to a health post in the village. They provide care and health education to the community both at the health posts and by outreach activities. Four of the HEWs interviewed in this study have obtained a diploma degree by completing an additional one-year training providing them with more knowledge on labour and delivery care.

The majority of clinical nurses and midwives complete a three-year diploma program which combines theory and clinical practice [28]. Their work responsibilities include identification and management of both normal and complicated deliveries. As the clinical nurses and midwives included in the study share similar backgrounds and responsibilities, they will for simplicity be referred to as nurse-midwives.

The research team had extensive discussions prior to the study about whether to include HEWs or not, as HEWs do not qualify as “Skilled Birth Attendants” according to the WHO definition. However, at the time of the initiation of the study, HEWs were mandated by Ethiopian policy to attend deliveries with no prior identified risk factors,^2^ and to refer complicated cases to higher-level health facilities (which however, is not always possible). HEWs were thus the communities’ first point of contact with the health system, and a most likely birth attendant to the majority of women. It was therefore considered relevant to assess whether the SDA could positively impact the quality of care and clinical outcomes at this level of care as well. However, during the course of the study, policy was changed, so that HEWs with only certificate level qualification were barred from conducting deliveries. This meant that the SDA became less relevant to a significant number of included health workers. However, in acute situations HEWs can be forced to help women in labour, and therefore, it was still relevant to include them in the study.

### Data collection

Qualitative data was collected in August 2013 (baseline) and approximately 1 year after the distribution of the SDA in October 2014 (follow-up) (see Table 1). At baseline, five focus groups discussions (FGDs) were conducted (*n* = 26) by AMFB with four groups of HEWs (*n* = 20) and one group of nurse-midwives (*n* = 6). The purpose of the baseline data collection was to explore how HEWs and nurse-midwives perceived their working conditions, including what they experienced as challenging and what they normally did to access information or instruction when they were in doubt about how to manage a complicated birth situation. At follow-up, six FGDs and four individual interviews were conducted by CFT. Four of the six FGDs were conducted with the intervention group (*n* = 23) of which three groups included HEWs and one group included nurse-midwives. Two FGDs were conducted with the control group (*n* = 10), of which one group included HEWs and the other group included nurse-midwives. Health workers from the control group were interviewed to examine whether the working experiences of the two groups diverted from each other and to assess to what extend the SDA is perceived to influence this.

**Table 1:** Participants and research activities at baseline and follow-up

	Job title	Educational level	Gender	Research activities	Total
Baseline participants	Midwife/nurse	4 Midwives	3 Women	1 focus group discussion	26
		2 Nurses	3 Men		
	Health extension worker	17 HEW (certificate)	20 Women	4 focus group discussions	
		3 HEW (diploma)			
Follow-up participants (Intervention)	Midwife/nurse	6 Midwives	4 Women	1 focus group discussion	27
		2 Nurses	4 Men	2 individual interviews	
	Health extension worker	17 HEW (certificate)	19 Women	3 focus group discussions	
		2 HEW (diploma)		2 individual interviews	
Follow-up participants (Control)	Midwife/nurse	2 Midwife	2 Women	1 focus group discussion	10
		1 Nurse	1 Man		
	Health extension worker	6 HEW (certificate)	7 Women	1 focus group discussion	
		1 HEW (diploma)			
Total		40 HEWs (certificate)	55 Women	11 focus group discussions	63^a^
		6 HEWs (diploma)	8 Men	4 individual interviews	
		12 Midwives			
		5 Nurses			

^a^Seven health extension workers from the intervention group participated in a focus group discussion at both baseline and follow-up. The total number of participants was therefore 56 health workers

The participants included in the FGDs were selected based on their interest and availability. Either a supervisor from Maternity Foundation contacted the health workers or they were asked to participate in an FGD when already gathered for other training sessions. To obtain detailed information on each subgroup and to minimize the risk of dominance and imbalance between the participants, FGDs were conducted with nurse-midwives and HEWs separately, and with intervention and control groups separately. However, a nurse-midwife participated in one group with four HEWs, as these five health workers were all present at the same training.

Mobile device data was used as secondary data and was captured using the programme QlikView. QlikView contains information on when and how much the SDA and its features have been used. This information was included in this paper as a supplement to understand the utilization of the SDA and whether differences in use exist between the different groups of health workers. It was also used to see if the health workers’ own perceptions of use follow their actual use patterns. The health workers participating in the individual interviews were also identified using this programme.

All interviews and FGDs were conducted in private rooms at or nearby the health centres. FGDs lasted from 1 h to 1 h and 40 min and individual interviews from 15 to 30 min. As the study participants mainly spoke the local language, Oromo, interviews and FGDs were carried out with two local interpreters working for Maternity Foundation.

### Data analysis

All interviews/FGDs were recorded and transcribed into English by the two interpreters. In order to ensure that everything was translated as correctly as possible, all transcriptions were compared with the audio files and field notes. If anything was unclear, the interpreters listened to the audio files again and corrected anything that was missing or imprecise. Data was analysed using Kirsten Malterud’s [29] systematic text condensation strategy as an inspiration. Systematic text condensation is a descriptive strategy focusing on the informant’s own perception of their experiences rather than investigating underlying meanings. This is linked to a phenomenological approach aiming to gain insight into the health workers’ lived experiences in the attempt to perceive the world from their point of view [29]. Hence, data was analysed inductively, using a bottom-up approach with a primary focus on the health workers’ experiences with the app and their perceptions of this tool. Patterns and themes were created through repeated readings of transcriptions and field notes reflecting both the objectives of the study and the topics raised by the participants. When visiting health facilities, the interpreter and moderator engaged in informal conversations with employees at the health facilities and with pregnant women waiting to deliver their babies. This provided the opportunity to get a better contextual understanding of the health workers’ work places and the facilities in which pregnant women give birth.

### Ethical considerations

Ethical clearance for the study was obtained in May 2013 from the Ethiopian Oromiya Regional Health Bureau. All health workers in the clinical trial were informed of the purpose of the study and that they at any time could withdraw from the study. Informed consent to participate was given by signature or fingerprint [25]. Interview/FGD participants were verbally informed of the content of the interviews/FGDs and that participating was voluntarily. It was emphasized that all information shared by the participants would remain anonymous and confidential. Before commencement of data collection, the participants were asked for consent to record what was said. An ID number was applied to the transcriptions in order to secure that the participants’ names and statements could not be associated. When referring to statements made by the informants in this article, work title and interview/FGD numbers have been applied in order not to reveal their identity.

## Results

### Perceived usefulness

All health workers described the SDA as a useful tool that provides important information and instructions on management of deliveries and obstetric emergencies, and thus, increases their knowledge and skills on how to manage normal as well as complicated deliveries. Health workers reported using the app in several ways, both as a reference while managing complicated deliveries or afterwards for reflections on provided care to identify if essential procedures had been missed.

“Before we got this app, we were not following procedure, but now this app makes us update our skills. And also, it helps as a reference, when you miss steps, because you can see what to do and redirect yourself” (Clinical nurse, FGD6, Follow-up).

Most informants across both groups of health workers stated that the app is useful as it is. Three HEWs suggested adding instructions to the existing videos on how to suture a tear, secure an IV-line and give fluid, and how to make episiotomy. Adding additional animation videos to the app was a recurrent request by both nurse-midwives and HEWs e.g. on how to manage antenatal care, postnatal care, obstructed labour and (pre) eclampsia as well as guidance on the identification of risk factors for complications during pregnancy^1^. However, the app occasioned a request from HEWs for drugs and equipment that they generally do not have access to and they felt they needed in order to be able to fully follow the directions of the app.

“In the app, there are many procedures, medical equipment and drugs like IV fluid and oxytocin. The equipment and medications are all useful to help mothers, who have complications of PPH. Also, there is a point that says ‘don’t refer a mother who is bleeding’. But as we don’t have IV fluid, cannula, oxytocin medicine or the skills to follow the procedures, the app orders us not to refer the bleeding mother, so what can we do? So it is my comment that it is good if we have the training and get the equipment as well as provision of the medications for our health posts.” (HEW, Interview3 with high use, Follow-up)

The quote shows that some health workers have limited skills and available material, and thus, do not feel capable to manage a complicated situation. To those, the app seems to accentuate an awareness of limitations. So although the SDA appears to empower health workers and give them confidence, the SDA makes shortcomings in terms of supplies and equipment more conspicuous.

Another general notion in informants’ statements was the description of the app as a practical tool that in a visual way shows how to manage normal and complicated deliveries - a kind of simulated clinical training that enhances their practical skills. It is perceived as a contrast to theoretical training, which the informants noted that they tend to forget because they are only rarely applying this theory in practice.

“Since I got this smartphone and app, I have experienced a great change in my personal skills. Before we got this app, what we had learned at school concerning deliveries was only theoretical. No practical application or experience. But now what we learn from this app is great. We can observe the procedures step by step and see the human anatomy” (HEW, FGD1, Follow-up)

Both nurse-midwives and HEWs mentioned that they used to fear that complications would occur, because of their lack of skills on how to manage these situations. Both groups reported that the SDA had diminished this fear and had led them to have a more positive perception of their work. This indicates the usefulness of the SDA on different levels of the health system.

### Usage of the app

Most health workers across both groups explained that they used the SDA either at home to update knowledge or while they were at work conducting deliveries. The informants using the app at work explained that they did so, either before a delivery when a pregnant woman came to the health post or while conducting deliveries, either in case a complication occurred or to ensure that everything was done in accordance with the application procedures. Some described using the app to guide co-workers, whereas others would turn on speaker volume in order to follow the steps of the app during a delivery. In emergency situations, the app was often used as a reference to see how to manage e.g. PPH and neonatal resuscitation or as a reminder of drug dosages and administration.

“Even though you have qualified to know the medication dosage, you may forget. For example, if PPH happens, you need to know whether the medication should be given as fluid, orally or as a muscular injection [ … ] When needing medication instructions, we used to refer to the books, but since we got this app, it helps us to remember the medication dosage and the medication instructions, and in emergencies, we use it as a reference in a simple way” (Clinical nurse, FGD6, Follow-up)

Some nurse-midwives and HEWs stated that they used the application often (e.g. every day, every other day or every 3–4 days), whereas others, mainly HEWs used it once or twice a week or only when the phone was charged. The QlikView data showed that the app was still used frequently though not as much as in the beginning of the study. The app was most often used during the day. However, a pronounced use of the app at night time also indicated that they used it while at work. Both nurse-midwives and HEWs described that they were reminded of watching the animation videos when receiving notifications. The content of these messages varied and were mentioned as a reason for watching all four videos.

Mainly HEWs experienced barriers of use including lack of access to charging the battery of the phone and the opportunity to assist only few deliveries (e.g. 1 per month). When power was unavailable, the HEWs had to travel to the district towns to charge their phones. In these situations, they only used the application briefly to check the information needed. Assisting few deliveries were only mentioned as a barrier by some of the HEWs with a certificate level, who perceived the SDA as less relevant to their work and therefore did not use it much.

”[ … ] it (the app) helps us in our work, but there are differences between the midwives and health extension workers. The reason why I am saying this is that the midwives are working with the needed medical and delivery equipment, but to us even though we have the movies on the app, we are mostly working to assist house to house deliveries and we don’t have the needed equipment” (HEW, FGD2, Follow-up).

Some HEWs mentioned their limited use explicitly, but it could also be observed at the FGDs, as the informants with limited use were quieter and did not provide many examples of use. A HEW with a low use of the SDA requested during one of the individual interviews that videos about antenatal care, diet, family planning and vaccination should be included, as this was more relevant to her work. It appeared throughout the FGDs that nurse-midwives and HEWs with a diploma level had used the SDA the most, as they were the ones predominantly providing examples of using the knowledge gained from the app in clinical situations. The QlikView data supported this, stating that nurse-midwives and HEWs with a diploma level, who participated in the qualitative study, had generally used the app twice as much as HEWs with only a certificate level. Despite the fact that HEWs with a certificate level had not used the app much clinically, they explained to have learned a lot and feel more confident in their work. Some HEWs used the app to provide health education to pregnant women informing them of possible complications and advising them to give birth at a health facility, whereas others had used the app in emergency situations when they did not know what to do and were unable to initiate a referral. Conclusively, the app was used in multiple ways depending on the specific health worker’s health structure, number of deliveries assisted, level of complication management as well as external factors such as frequency at health structure, time available and power situation.

### Clinical practice before and after SDA implementation

In general, both nurse-midwives and HEWs described significant improvements in their clinical practice when comparing their clinical practices before and after the distribution of the SDA. Previously, in situations where HEWs were not able to assist a pregnant woman, they would contact the health centre or a private clinic in order to see if anyone would be able to come to the health post to assist.

“There was a time where a baby was full of mucus and was floppy. I didn’t have a suction to clean it and I therefore used a syringe without needle. But it didn’t clean properly. Fortunately, there was a nurse with me who came to give support. [ … ]. If there hadn’t been someone with me that baby would have died, as I don’t know what to do” (HEW, FGD5, Baseline)

If HEWs were not able to get help from other health workers, they would have to manage the best they could or refer the pregnant woman to a higher-level health facility. However, both nurse-midwives and HEWs described at baseline how the referral link is weak with lack of ambulances and accessible roads between health posts and health centres:

“[ … ] what makes me hate my job is that I teach my community that mothers should not give birth at home, but that they should come to health post to be assisted by us. If we can’t help, we’ll call an ambulance, which will take the mother to the next health facility. But one time I called an ambulance and they said ‘we are coming, let the community bring the mother half way’, mentioning the place to meet. The community was willing to do so. However, the ambulance did not come and they had to carry the mother all the way to the health centre. When this happened, I felt like I was convincing the community on false service, which can’t be given to the community. I tried to convince my community about what had happened and asked for excuse, but the community was sad at that particular time” (HEW, FGD1, Baseline)

Some of the nurse-midwives also described how they previously initiated referrals in case of complications:

“Because the hospital is located nearby our health centre, if we observe any bleeding and have doubts about what to do, we fear and immediately initiate a referral to the hospital, because we don’t have confidence and don’t have experience on controlling PPH. But now, we have confidence and no longer fear because we have gained more knowledge from the Safe Delivery App” (Midwife, FGD6, Follow-up)

Nurse-midwives have experienced that the app has helped them to recollect and revise their skills, which have made them better capable of managing clinical situations in accordance with the recommended procedure. HEWs, in contrast, had a very limited knowledge of management of deliveries and had thus gained much new knowledge from the app, including the reasons for complications and explanations about how to solve them:

“I remember one time after we got the training and the Safe Delivery App from the organization, a woman was bleeding a lot because of an abortion. When her family called me on the phone, I was working far from the health post. During that time I hadn’t seen the app video in detail. Because I don’t have much skill and didn’t see the app in detail, I informed her parents to immediately take her to the health centre. Then when I saw the Safe Delivery App, I was surprised. While I have misoprostol at the health post, I sent her to the health centre, which made me feel sad and ashamed of myself. But now since I got this app and have seen it in detail, I am helping every woman who come to the health post for treatment” (HEW, FGD2, Follow-up)

HEWs described how they have improved their knowledge on how to care for mothers and newborns by stimulating the newborn and ensuring attachment, checking the pulse and blood pressure and giving the mother misoprostol before sending her home. In addition, they described to have learned how to suck meconium from the newborn and how to conduct resuscitation. In case of PPH, they most often used the app to identify drugs and dosage to be given.

“Even if I know that by giving an injection, bleeding can be stopped, I learned that giving 600mg^3^ (sic) misoprostol tab orally will stop the bleeding. I didn’t know that by massaging the uterus, the uterus can be contracted, but now I have learned from the app that massaging the uterus will make the uterus contract and stop the bleeding” (HEW, FGD1, Follow-up)

Especially HEWs described that their lack of knowledge and the need for essential drugs meant that their previous practice led to a high risk of maternal mortality. One of the significant changes expressed by HEWs was that they had learned the importance of stopping the bleeding before referring the mother to a higher health facility in case of PPH. Despite the lack of essential drugs, the HEWs in this way described the SDA as a tool that has helped them bring about change in their perception of own skills and knowledge. Most nurse-midwives stated that they were now able to manage PPH and retained placenta at the health centre, whereas they previously would have referred the woman to the hospital if the situation became serious. The SDA teaches nurse-midwives and HEWs the lifesaving steps of how to stop a bleeding by performing uterus massage, applying bimanual compression and providing essential drugs. Though HEWs were taught how to stop a bleeding, they were, however, not capable of e.g. suturing a tear or provide IV fluid and would therefore in serious situations still have to refer women in labour to a higher health facility.

### Community reactions

According to both nurse-midwives and HEWs, the quality of care, which the community experience at the health facilities influence the level of trust in their services. After receiving the SDA, both groups claimed to have gained increased recognition and trust from their communities. Especially the HEWs who prior to receiving the app declared a high degree of distrust from community members felt that this had changed remarkably.

“There was a time where a woman in labour came to our health post together with her husband. As they reached the health post, the husband starts requesting us to call the ambulance from Gimbie town and tells us not to touch her. At that time, we had this Safe Delivery App. I said to her husband that I should check his wife first then based on her situation I would call an ambulance, if needed. But he refused and said ‘Because I have lost a wife and others have lost their wives in such a place, I do not want her to be delivered here’. He continued requesting us to call an ambulance. Then we just handled the case normally and convinced him to let us examine the woman. If she had any complications, we would call the ambulance. But when we observed, we saw that she was in normal condition and therefore she was delivered safely at our health post. By watching the Safe Delivery App, we supported and treated her accordingly.^4^ Then the husband was so happy. He appreciated and blessed us and took her home safely. Therefore, by using the app our confidence has developed and the community gives encouraging feedback” (HEW, FGD1, Follow-up).

Most community members are said to not know about the app, but they see how the skills of health workers have changed and consequently inform others about the enhanced service they get at the facilities.

“I can say that there is a great change at the health posts and in our skills too. It develops our trust in the community. So in our Kebele, the number of women being delivered at my health post has increased. For example in one month, I assist three delivery cases, sometimes more. This change has happened after a woman came with labour. Until the baby was delivered, I was watching the Safe Delivery App movie in the local language and while examining the woman I made the volume loud. All the people who had come with her were listening to what the app said. After the baby was safely delivered, the woman and her family went home and informed the community that our skills had changed and that we have a supportive tool to assist pregnant women.” (HEW, FGD1, Follow-up).

As a result of these improvements, both nurse-midwives and HEWs have experienced co-workers expressing interest in the app and doctors at the Government Hospital, had asked to get the app transferred to their phone:

“Because we have shown great change in our work and updated ourselves on delivery cases, our confidence has increased. The staff, we work with wish to have the Safe Delivery App and develop their skills on conducting deliveries. They are so happy to see how we have changed, and they would like to receive additional training” (Midwife, FGD6, Follow-up).

## Discussion

The findings indicate that the SDA is perceived by its users as a useful tool, which strengthens their skills and knowledge. They use it both for education to improve and retain knowledge and skills, and as an emergency tool when immediate guidance is required to manage obstetric and neonatal complications. Most health workers feel that the SDA has improved their confidence and ability to manage childbirth situations, which they were previously insecure about. These qualitative findings are supported by the cluster-randomized clinical trial, which shows that health workers in the intervention group have increased their knowledge and skills in neonatal resuscitation more than 2-fold compared to the control group 6 and 12 months after introduction to the SDA [25].

The perceived usefulness of the SDA seems to be related to several parameters. One is that the tool is experienced to be practical in its instructions, and helps its users translate theoretical knowledge into practice, by visually communicating which steps to take. Nilsson et al. [26] similarly reported, in a small study of 27 Kenyan nursing students, that a non-interactive training video, which showed how to manage PPH using role-play scenarios, appeared to be as effective as interactive hands-on training. Thus, mHealth tools providing visual information and instruction might be a potential solution to improve knowledge and skills of health workers with particular relevance to health workers serving in remote areas.

The voice-over in local language was another parameter that health workers found useful. They described turning the SDA on speaker volume when complications occurred and they needed guidance, using the app interactively. In a study of Ugandan nurses, Martin [30] described the embodied employment of technological tools by providing examples of how a syringe or a thermometer became an extension of the nurses’ hand, as these were unreflectively integrated in the performance of specific tasks. For some of the health workers, the app seems to have similarly become indispensable in complicated delivery situations and can be portrayed as an ‘extended hand’ helping them to do something in situations they cannot manage otherwise.

The improved competencies and the access to an ‘emergency tool’ have let both HEWs and nurse-midwives to feel more empowered to conduct their work because they perceive to have obtained more skills and knowledge. Other Ethiopian studies investigating the effect of other smart phone interventions have similarly found high acceptance levels and an increased sense of empowerment among its users [18–20]. Krubiner et al. reviewed 94 health programs to examine how nurses and midwives can be empowered [31]. The review concluded that nurses and midwives, who have access to continuous training and skill development experience greater autonomy and self-confidence, which can lead to improved quality of care delivery and respect from peers and communities [31]. The respect from the communities, which the health workers describe to experience, is likely to both result from the health workers’ ability to deliver better care, but may also result from the ‘symbolic value’ and authority connected to the ownership of technological tools [30, 32].

The high level of user acceptance combined with the documented impact of the SDA is interesting considering the gap of ensuring access and adherence to evidence based clinical guidelines in low- and middle-income countries. This problem has been demonstrated in a recent survey among 69 member associations of the International Federation of Gynaecology and Obstetrics, which showed that there is a lack of comprehensive, up to date evidence-based national guidelines on PPH [33]. Almost 20% of the associations do not have national guidelines on PPH management and among those who do, guidance on how to use misoprostol is often absent [33]. Furthermore, a review by Miller et al. emphasizes the need to ensure implementation of and adherence to evidence based clinical guidelines for routine maternal health care and calls for a global approach to ensure quality and equitable maternal health [34]. mHealth tools such as the SDA, which ensures access to the most recent evidence based clinical guidelines, and is demonstrably very accepted by its end-users, may help bridge this gap.

Another finding was, that the SDA seems to have the potential to both empower and spur health workers, working under the difficult circumstances in a weak health system, to be able to see, accentuate and formulate their limitations into concrete demands for adequate training and essential equipment and drugs – while the app at the same time also empowers them to do more with what they have, e.g. their own hands, or the drugs they have available. This was pronounced in statements from the included HEWs, who have only received limited educational training on clinical management of normal deliveries, which they, at the time of the study, were responsible for managing, whereas women with complications should be referred. However, due to a weak referral system HEWs are in reality often the sole provider present when complications occur, which left many HEWs feeling under pressure, aware of their professional limitations in caring for these women. A too strong focus on the structural limitations could also pose the risk that some health workers’ miss out on the opportunities that the app empowers them with; namely what they actually can do with their bare hands, e.g. the opportunity to conduct a bi-manual compression and control the bleeding that way.

The app was used in multiple ways. Statements from nurse-midwives, who have more skills and confidence, showed, that they primarily use the app to revise, refresh and retain their skills. Nurse-midwives and HEWs who conduct many deliveries more often state that they also use the app in clinical situations compared to those who only conduct few deliveries. Hence, user patterns follow the relevancy of the tool to the health workers’ work situation, and thus, the app is used in multiple ways depending on work context, practical cases, level of frequency at health structure and other contextual factors.

The following study limitations have been considered: as the interpreter and moderator were both known to be associated with Maternity Foundation, which is one of the implementing agencies, there is a risk that the informants’ responses might have been influenced to gravitate towards positive examples of the SDA use, out of politeness. In order to reduce this risk, it was emphasised to informants that the researchers would like to learn from both positive and negative experiences and any experience they could share was welcome. In addition, data was triangulated, using multiple data sources, to confirm the statements of informants, e.g. talking to the participants both individually and in groups, observing facial expressions and body language, the way they interact, and the conditions under which they work. As the FGDs were carried out with homogenous groups, there is a risk of response bias with some informants agreeing with others in order not to stand out. However, as the informants often provided different examples of use, this did not seem to be a concern. For instance, some HEWs described a very limited use because of lack of electricity or few deliveries at the health posts.

## Conclusion

The health workers perceive the SDA as a useful tool, which helps them update and revise their knowledge and skills, increases their confidence and makes them feel better able to deliver quality care. Consequently, they experience more trust and respect from the communities they serve. The positive perceptions of the SDA suggest that mHealth tools providing visual learning videos may be a potential way to improve knowledge and skills of health workers serving in remote areas. Being perceived as a relevant and useful tool, the specific perception and use of the SDA is multiple, embedded in and contingent upon the specific work situation of the health worker. Consequently, midwives and other skilled health workers has a high practical level of use of the SDA, whereas the SDA among especially HEWs is helpful in strengthening skills and confidence, while at the same time zooming in on structural limitations, aiding them in advocating for a strengthened enabling environment.

## Abbreviations

FGD: Focus group discussion
HEW: Health extension worker
mHealth: Mobile phones for health
PPH: Postpartum haemorrhage
SDA: Safe Delivery App

## Footnotes

1 Informed by user feedback and other inputs, the Safe Delivery App has since the study been further developed into a version that contains ten films that cover all the seven signal functions of Basic Emergency Obstetric and Newborn Care as well as films on essential preventive measures. The additional videos include post abortion care, hypertension, prolonged labour, maternal sepsis, newborn management and infection prevention. This version of the app was launched in April 2015 and has since then been made accessible and free for download (https://itunes.apple.com/us/app/safe-delivery/id985603707?mt=8 or https://play.google.com/store/apps/details?id=dk.maternity.safedelivery)

2 This, however, is not always possible, as most complications cannot be predicted and happen to “low risk” pregnant women. This may have been one of the reasons why a political change was later made withdrawing health extension workers’ mandate to deliver women.

3 The app teaches to use 600 mg. preventively and 800 mg. to treat the bleeding.

4 The Safe Delivery App contains the video Active management of third stage of labour, which explains how to prevent post partum haemorrhage.
